# Development of a hard X-ray delay line for X-ray photon correlation spectroscopy and jitter-free pump–probe experiments at X-ray free-electron laser sources

**DOI:** 10.1107/S0909049511004511

**Published:** 2011-03-19

**Authors:** Wojciech Roseker, Hermann Franz, Horst Schulte-Schrepping, Anita Ehnes, Olaf Leupold, Federico Zontone, Sooheyong Lee, Aymeric Robert, Gerhard Grübel

**Affiliations:** a Hamburger Synchrotronstrahlungslabor am Deutschen Elektronen Synchrotron (DESY), Notke­strasse 85, D-22603 Hamburg, Germany; b European Synchrotron Radiation Facility, 6 rue Jules Horowitz, BP 220, 38043 Grenoble Cedex 09, France; c SLAC National Accelerator Laboratory, 2575 Sand Hill Road, Menlo Park, CA 94025, USA

**Keywords:** delay line, XPCS, pump–probe, X-ray optics

## Abstract

A hard X-ray delay line capable of splitting and delaying single X-ray pulses has been developed with the aim of performing X-ray photon correlation spectroscopy (XPCS) and X-ray pump–probe experiments at hard X-ray free-electron laser sources. The performance of the device was tested with 8.39 keV synchrotron radiation. Time delays up to 2.95 ns have been demonstrated. The feasibility of the device for performing XPCS studies was tested by recording static speckle patterns. The achieved speckle contrast of 56% indicates the possibility of performing ultra-fast XPCS studies with the delay line.

## Abstract 2

A prototype device capable of splitting an X-ray pulse into two adjustable fractions, delaying one of them with the aim of performing split pulse X-ray photon correlation spectroscopy and pump–probe type studies was designed and manufactured. Time delays up to 2.95 ns have been demonstrated. The achieved contrast values of 56% indicate a feasibility of performing coherence-based experiments with the delay line.

## 1. Introduction

Probing matter on time scales ranging from femtoseconds to nanoseconds is one of the key attributes offered by X-ray free-electron lasers (XFELs) (Altarelli *et al.*, 2006 ▶; Patterson *et al.*, 2010 ▶; Shintake *et al.*, 2008 ▶). These new X-ray sources provide ultra-short and coherent pulses that carry more than 10^12^ photons per pulse. Apart from the extremely high peak brightness and coherence, the most prominent feature of the XFEL machines is their time structure. The European XFEL will deliver sub-100 fs-long pulses separated by 200 ns in bunch trains arriving with a repetition rate of 10 Hz (Altarelli *et al.*, 2006 ▶). The Linac Coherent Light Source (LCLS) will provide sub-100 fs pulses at a repetition rate up to 120 Hz (Emma *et al.*, 2010 ▶).

Such capabilities of the new light sources will enable new types of experiments currently not feasible with third-generation storage rings. However, the intrinsic time structure of the machines and the timing jitter between pump and probe beams need to be addressed to fully utilize the ultra-short pulse duration. For instance, in X-ray pump–X-ray probe or X-ray photon correlation spectroscopy (XPCS) experiments (Sutton *et al.*, 1991 ▶; Grübel & Zontone, 2004 ▶) the shortest time scale that can be measured is set by the minimum pulse spacing and the pulse duration of the source. Therefore, employing the intrinsic time structure of the XFEL source in XPCS or X-ray pump–X-ray pump experiments will limit the access to time scales shorter than 200 ns at the European XFEL or 8.3 ms at LCLS.

Adapting the time structure of the XFEL source to the needs of the aforementioned experiments can be achieved by split pulse and delay techniques (*i.e.* usage of a delay line). Moreover, the pulses provided by a delay line (*i.e.* the main pulse and its delayed replica) originate from the same electron bunch and thus give an intrinsic synchronization to the X-ray source. Furthermore, the time resolution of the pump–probe experiment is determined not by a detector but the delay line itself. In ultra-fast XPCS experiments (Grübel *et al.*, 2007 ▶; Gutt *et al.*, 2007 ▶) it provides an intrinsic normalization of the measured speckle pattern contrast. In this case, any bunch-to-bunch instabilities (*e.g.* change of intensity or coherence properties of pulses) coming from the XFEL source will not affect the experiment. In addition, the use of a delay line in XPCS puts less demands on the detector performance. The time resolution of the detector used in the experiment must be only better than the minimum pulse spacing of the X-ray source.

Existing delay lines operate in the spectral range from visible radiation to soft X-rays (Mitzner *et al.*, 2005 ▶; Sorgenfrei *et al.*, 2010 ▶). Equivalent devices for hard X-rays have been discussed for many years and several beam geometries have been proposed (Altarelli *et al.*, 2006 ▶; Arthur *et al.*, 2002 ▶; Materlik & Tschentscher, 2001 ▶). We have recently reported on the first successful tests of a hard X-ray delay line (Roseker *et al.*, 2009 ▶).

In this paper we demonstrate the operation of the X-ray delay line at 8.39 keV. The device provides access to time scales below 2.95 ns, opening up new possibilities for XPCS and pump–probe experiments at XFEL sources.

## 2. Conceptual layout

**Figure 1:**
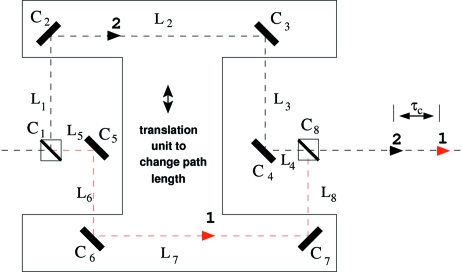
Schematic layout of the X-ray delay line. Eight optical components are arranged in 90° scattering geometry. 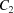, 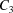, 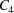, 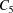, 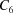, 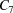: Bragg reflectors. 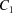: beam splitter. 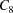: beam mixer. 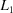, 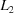, 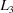, 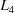, 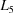, 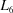, 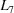, 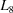: path lengths inside the delay line.

The concept of the delay line is based on single-crystal diffraction used to split an X-ray pulse into two parts and recombine them with a time delay. Fig. 1 ▶ shows a sketch of this concept. The incoming X-ray pulse is first split by a beam-splitter crystal 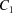 into two pulses, that propagate along two unequal rectangular paths. The optical path (hereafter called ‘upper branch’) for one of the split pulses is defined by the Bragg crystals, denoted subsequently 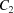, 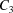 and 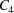. The other split pulse is guided *via* the Bragg crystals, 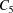, 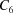 and 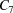 (hereafter called ‘lower branch’). Both pulses are brought back on a common path at the beam-mixer position 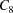 and propagate in the same direction.

The delay 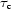 between the arrival time of the two split pulses at the sample position is defined by
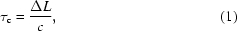
where 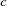 is the speed of light. The path length difference 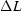 is the difference between the path lengths of the upper and lower branch, *i.e.*
            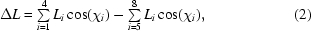
where 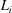 and 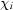 are path lengths inside the delay line (as indicated in Fig. 1 ▶) and the tilt of the corresponding 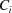 crystal, respectively. Owing to the 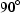 geometry one finds that the beam paths 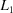 = 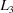 and 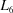 = 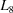. Moreover, the path lengths 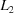 = 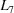 and 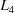 = 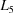. When the beam is aligned exactly in the vertical scattering plane, *i.e.* 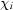 = 0, the path length difference simplifies to 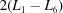.

A change of the path length difference 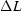 and delay time 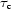 is achieved by simultaneous vertical movement of the crystals 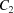, 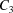, 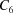, 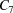 in the direction perpendicular to the incident beam, as shown in Fig. 1 ▶. Since the concept employs upper and lower delay branches, the minimum delay time accessible by the set-up is not compromised by the size of the employed optical components.^1^ Moreover, usage of the two delay branches allows accessing 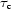 = 0 for time calibration purposes.

**Figure 2:**
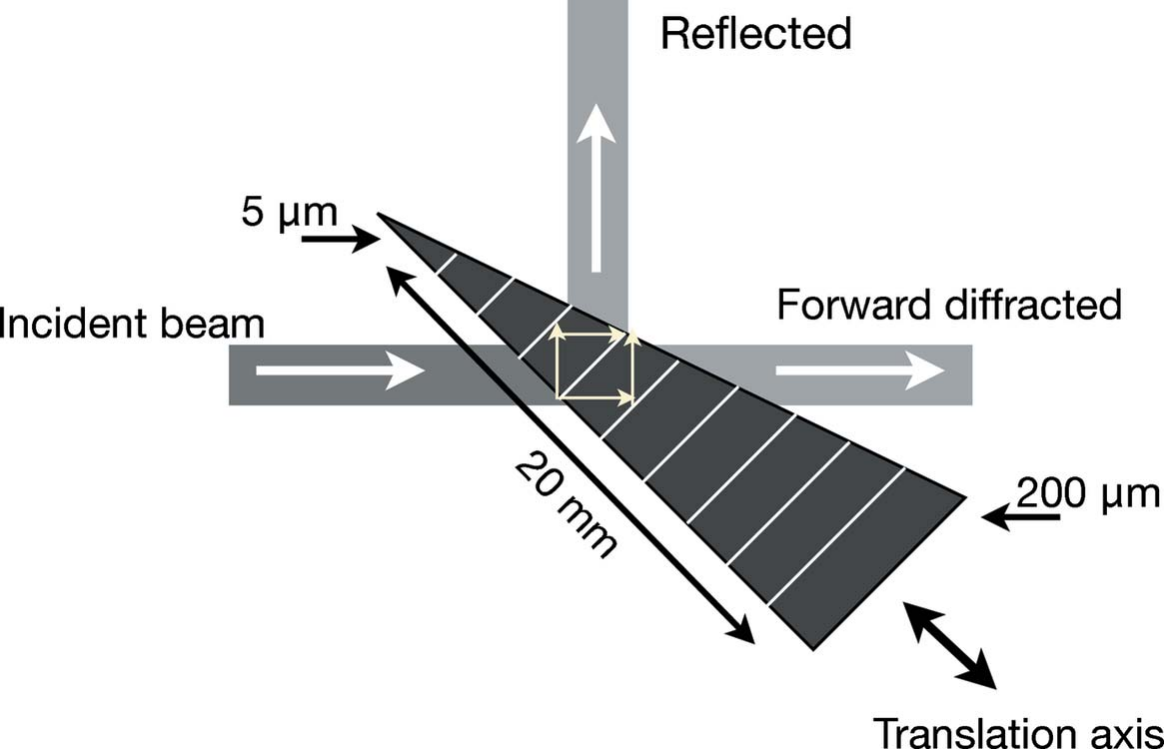
Schematic layout of the delay line X-ray beam splitter. The crystal is oriented in Laue diffraction geometry. By moving the crystal along the translation axis, different thicknesses of the crystal are probed.

**Figure 3:**
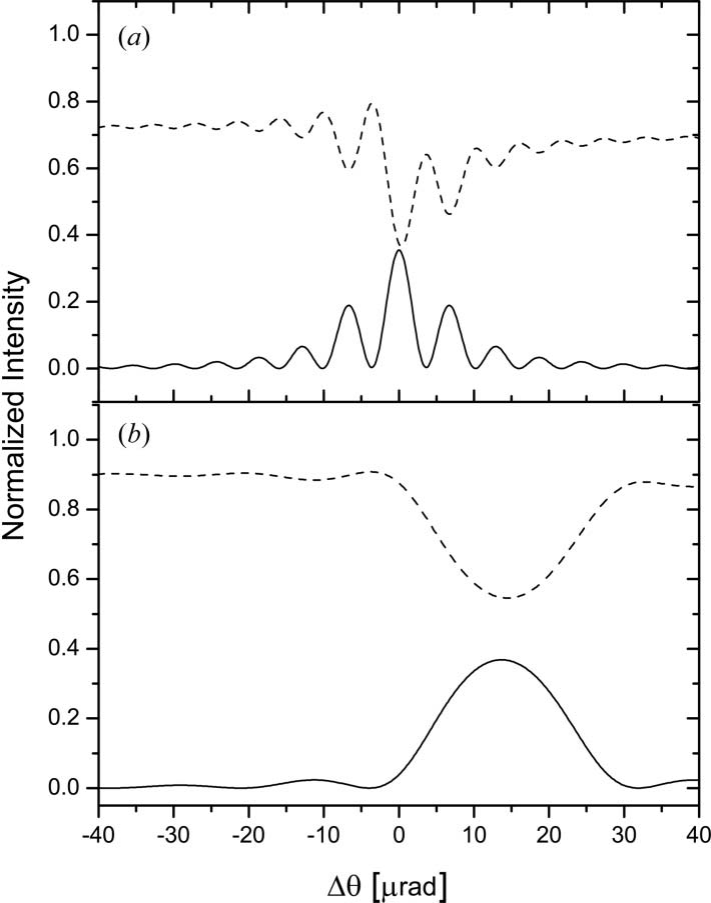
Variations of reflected 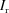 (solid line) and forward-diffracted 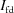 (dashed line) intensities as a function of 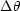 = 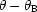, where 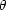 is the incident angle and 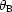 = 45°. Calculations were performed using the *XOP* 2.11 package (Dejus & Sanchez del Rio, 1996 ▶) at *E* = 8.39 keV for a symmetric Si(511) oriented in (*a*) Laue geometry with a crystal thickness 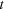 = 19 µm and (*b*) Bragg geometry with 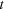 = 6 µm.

The splitting and recombination of X-ray beams inside the delay line is accomplished by perfect thin crystals oriented in Laue geometry. Fig. 2 ▶ shows the schematic layout of the Laue beam splitter. In order to tune the ratio of the pulse power between the split beams the splitter has the form of a wedge. With an apex angle of 10 mrad and height of 20 mm, the thickness of the crystal, illuminated by the X-ray beam, can be continuously varied from the top to the bottom by moving the crystal along the translation axis, as depicted in Fig. 2 ▶. The splitting capability of the crystal depends on the ratio of the beam-splitter thickness 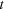 to the Pendellösung period 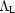 (Authier, 2001 ▶). Probing the crystal at a thickness corresponding to 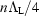, where 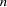 is a positive odd number, yields equal intensity splitting. For instance, at *E* = 8.39 keV the Si(511) reflection yields a Pendellösung length^2^ 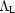 of 25 µm. A splitting ratio close to 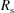 = 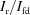 ≃ 1 can be achieved at a thickness of 3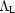/4 = 19 µm, as shown in Fig. 3(*a*) ▶.

Combining Laue and Bragg optics of the same index of reflection in the delay line slightly modifies the exact 90° scattering geometry affecting the incident beam conditions for the experiment. In Bragg geometry the center of the reflection domain deviates by 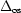 from the kinematical Bragg angle owing to the refraction effect. In symmetric Laue geometry, 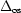 = 0. As a consequence the two delayed X-ray beams exit the delay line with a vertical angular mismatch 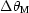 = 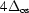. Using Si(511) Laue and Bragg optics at *E* = 8.39 keV leads to 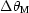 = 54 µrad. At higher energies the refraction effect is weaker, *e.g.* *E* = 12.4 keV the mismatch is 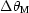 = 25 µrad. The angular mismatch between the delayed beams can be of advantage for X-ray pump–X-ray probe experiments, creating a possibility to separate the background of a strong pump beam from the signal of the probe beam. The co-linearity of the two beams is, however, very important in XPCS experiments in order to ensure that the same sample volume is illuminated by the two delayed beams.

The angular mismatch can be compensated in several ways:

(i) By varying the temperature of the Laue crystals by 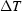 = 0.5 K.

(ii) By tilting the crystals 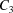, 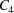, 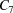 and 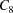 out of the scattering plane.

(iii) By employing a Bragg beam splitter and mixer.

Fig. 3(*b*) ▶ shows intensities 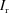 and 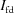 as a function of relative incident angle 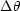 calculated for a thin Si(511) Bragg crystal. A splitting ratio 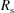 close to 1 is achieved using a 6 µm thin crystal. As shown in Fig. 3 ▶, in order to equally split the beam at 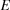 = 8.39 keV the thickness of the beam splitter has to be much smaller than in the Laue case. This causes constraints for the crystal manufacturing process. It should be noted, however, that a Bragg crystal with a thickness larger than 6 µm can be used for beam splitting as well if one were to accept slightly different energies for the two delay branches. In this case the crystal acts as a Bragg mirror for the selected energy. The radiation outside the Bragg bandwidth is efficiently transmitted.

## 3. Experimental set-up

### 3.1. Design requirements

To implement the concept described in Fig. 1 ▶ the delay line must meet several conditions.

(i) The device employs single crystals to split and delay the X-ray beam. Thus, the angular resolution of the involved mechanical components of the delay line optics must be higher than the width of the crystal reflections.

(ii) The mechanical design of the device must provide delay time tunability. Ideally, the delay time should be adjustable without complicated procedures including crystal realignment. The delay time is adjusted by means of a simultaneous translation of the four crystals (*i.e.* 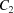, 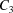, 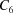, 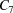, shown in Fig. 1 ▶). The most straightforward way to do that is to place all four crystals on a single translation stage. In this case the maximum delay time and time resolution depends on the range and accuracy of the single translation stage. However, one should note that any mechanical inaccuracy causing deviation from the straight motion of the translation will result in a loss of reflectivity and the necessity to realign the delay line optics.

(iii) To ensure mechanical stability of the set-up the design is based on a massive granite support.

(iv) For XPCS studies the two delayed beams that exit the delay line should co-propagate in the sample direction. For pump–probe experiments the set-up should allow the angle between the pump and probe beam paths to vary.

(v) The overall dimensions of the delay line are of importance as well. The device should fit in the experimental hutches at various beamlines allowing performance tests at various sources to be carried out. Therefore, the size of the device and maximum achievable delays should be optimized.

### 3.2. Mechanical design

**Figure 4:**
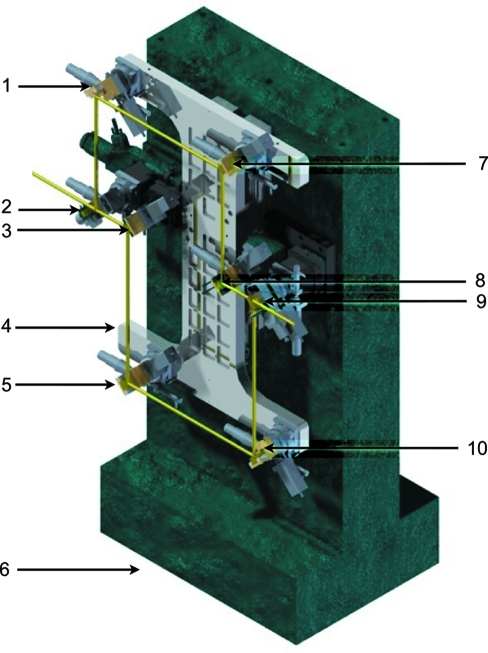
Three-dimensional model of the delay line. (1), (3), (5), (7), (8), (10): Bragg reflector stages. (2): Beam splitter stage. (9): Beam mixer stage. (4): Aluminium plate. (6): Granite support. The X-ray beam path is indicated by the yellow line.

Fig. 4 ▶ shows the design of the X-ray delay line. The set-up consists of a massive 380 kg granite support (6) and five main subassemblies: central translation unit, beam splitter assembly (2), beam mixer assembly (9) and two Bragg crystal holders (3), (8). For stability reasons all four crystal holder assemblies are mounted directly on the granite support (6). The central translation unit consists of four Bragg crystal holders [*i.e.* (1), (5), (7), (10)] mounted on an aluminium plate (4). The plate is mounted on a custom-made high-precision translation stage (HUBER 5101.30-300hp), which is fixed to the massive granite support (6). Since the X-rays from XFEL sources are linearly polarized in a horizontal plane, all optical components diffract the beam in the vertical scattering plane.

In order to optimize the delay time adjustment, the set-up works in a fixed 90° scattering geometry. In this case a single translation stage is sufficient to change the path length difference 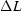 inside the delay line. The maximum range of the translation stage is 300 mm. This gives the maximum accessible path length difference 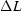 and delay time of 798 mm and 2.66 ns, respectively. The resolution^3^ of the translation stage is 0.5 µm and sets the resolution of the delay time to 7 fs in this configuration. Access to longer time delays can be provided by removing the crystals 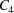 and 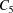 out of the beam path and placing the beam splitter on the position 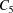. In this case the delay line operates only with the lower branch in so-called pump–probe mode with a minimum delay of 2.5 ns. In this configuration the maximum delay time and time resolution are 3.5 ns and 3 fs, respectively.

In order to reduce temperature drifts acting on the delay line optics the set-up is enclosed by a Plexiglass box. Additionally, a support construction was designed, providing suitable translations and rotations for the alignment of the granite support to the X-ray beam. The horizontal translation, with a travel range of 50 mm, allows the delay line to be moved in and out of the incident X-ray path. The support structure comprises three adjustable feet allowing to correct for tilts of the granite support. The size of the whole assemble is 930 × 990 × 1990 mm.

### 3.3. Crystal optics

**Figure 5:**
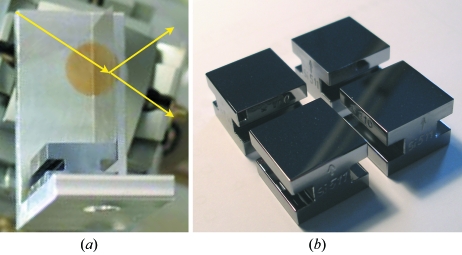
(*a*) Laue crystal beam splitter. Beam geometry is denoted by the yellow line. (*b*) Four Bragg crystal reflectors.

Owing to the employed fixed-angle scattering geometry, the energy at which the delay line is operated can be varied only in discrete steps. This is achieved by selecting appropriate crystal reflections with 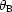 = 45° at the given energy. The choice of crystal reflections spanning the energy range from 8.39 to 12.914 keV is given in Table 1 ▶. To perform experiments at 8.39 keV a set of perfect silicon (511) crystals was fabricated from a high-quality ingot and cut in Laue and Bragg geometries. The crystals have a reflectivity of *R* = 0.89 and Darwin width of 8.6 µrad. Figs. 5(*a*) and 5(*b*) ▶ show the beam splitter and Bragg crystal reflectors. The beam-splitter crystal was cut into a wedge form and etched in the solution of HF, HC_3_COOH and HNO_3_. Although each beam splitter was prepared according to the same etching procedure, each crystal has slightly different thickness variations. The deviation of the apex angle from crystal to crystal is, however, less than 1.74 mrad. The crystal foot has grooves on both sides, allowing it to be clamped to a holder in a stress-free way. Typical dimensions of the splitter are given in Fig. 2 ▶.

To maintain the reflection conditions at 8.39 keV the experimental set-up was equipped with very high precision mechanics. High angular resolution was achieved with piezo actuators (PI M-035), mounted on each crystal stage. To align the beam to the scattering plane all crystals were placed on tilt stages (KOHZU SA04B-RT).

### 3.4. Delay line controls

The alignment of the delay line crystal optics and the change of the delay time are fully controlled by a PC. The communication between the computer and the device is realised *via* VME electronics. This includes motor controllers (OMS VME58) and SINCOS power supplies for 32 motorized devices (16 translations, 8 swivel and 8 rotations stages). Each stage is equipped with a micro-stepping option, which improves its resolution by a factor up to 20. Eight piezo low-voltage actuators are controlled by LVPZT controllers (E-621) that are installed and networked in one chassis. The connection between the chassis and the PC is established *via* RS-232.

### 3.5. Delay line diagnostics

The diagnostics system of the delay line is based on two detector types: ionization chamber (IC) and avalanche photodiode (APD). Nine ICs are distributed inside the delay line, placed between each pair of crystals. The use of IC detectors allows the crystals to be aligned to the Bragg angle. In addition, each chamber monitors the intensity inside the delay line. To visualize the beam position along the delay path a set of removable fluorescence screens is mounted on the aluminium plate.

**Figure 6:**
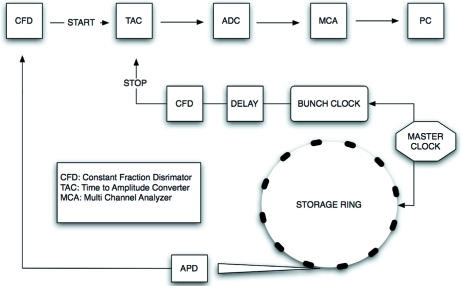
Signal processing of the time delay measurements.

Time delay measurements are performed with an APD detector (Kishimoto, 1991 ▶; Baron *et al.*, 2006 ▶). The detector consists of a silicon diode and a fast amplifier. It is mounted in a metal case to reduce possible noise and reflections. The front side of the detector is covered with a thin aluminized mylar window, used as a shielding against parasitic light. Fig. 6 ▶ shows a schematic of the electronics used in time delay measurements. The detector signal is amplified close to the diode by a fast pre-amplifier. The signal is sent with an amplitude of typically 20 to 100 mV to a constant fraction discriminator (CFD; Ortec 935), which filters out the noise from the amplifier. The output signal from the CFD is used as a start trigger of a time-to-amplitude converter (TAC; Ortec TAC/SCA 567). The bunch clock signal from the synchrotron ring is used as a stop pulse for the TAC. Since the TAC range is limited, the arrival time of the stop pulse can be adjusted by an electronic delay unit. The TAC produces a pulse with an amplitude proportional to the time interval between the start and stop pulses. The amplitude distribution of TAC pulses is digitized by an analog-to-digital converter (ADC; Canberra 8715) and stored in a multichannel analyzer (MCA; TVME200). The conversion of channels to time is determined with the time calibrator (Ortec 462) or the electronic delay unit.

## 4. Performance of the delay line

The delay line is a tool for conducting pump–probe and correlation spectroscopy experiments. To utilize this device in these techniques it is necessary to understand the behavior of each optical component and quantify its performance. The X-ray delay line utilizes two Laue and six Bragg crystals, that are arranged in dispersive (+*n*, +*n*) and non-dispersive (+*n*, −*n*) configurations. To characterize the throughput of such a complex optical system experiments are performed in steps. First, the quality of the Bragg optics in the four crystal Bragg configuration is investigated. Then the splitting performance of the X-ray beam splitters is verified. Finally, the throughput and the stability of the delay line with the Laue beam splitters is measured in the two-branch configuration.

The performance tests have been carried out at DORIS III, PETRA II (Hamburg, Germany) and the European Synchrotron Radiation Facility (Grenoble, France). All experiments were performed at 8.39 keV with a relative bandwidth of 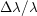 = 8.6 × 10^−6^. The initial tests of the delay line using the hard X-ray radiation were carried on the C and W1 beamlines at DORIS III. Synchrotron pulse delay experiments and coherence-based experiments with the undulator source were conducted at beamline ID10C at ESRF. In this section we briefly summarize all these experiments.

### 4.1. Bragg optics

**Figure 7:**
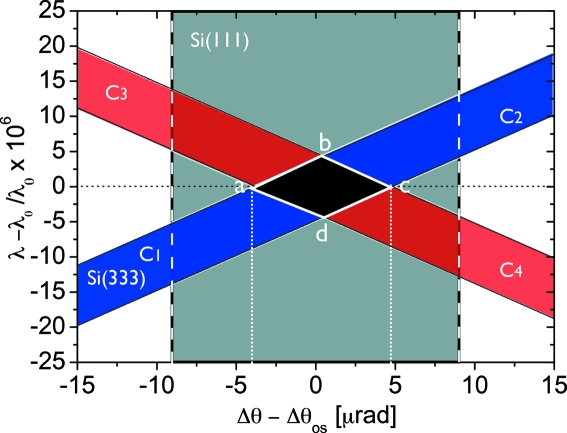
DuMond diagram of the Bragg crystal configuration of the delay line. The incident and exit divergence is represented by dashed and dotted lines, respectively.

To observe an almost intrinsic rocking-curve profile one needs to employ an extremely collimated and monochromated X-ray beam (Ishikawa *et al.*, 1991 ▶). This condition implies the use of crystal monochromators and beam collimating optics. Both can be achieved by using asymmetric reflections. However, even without the use of asymmetric reflections, information on the quality of each optical element can be obtained. Since the crystals 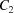 and 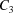 are arranged in dispersive configuration (+*n*, +*n*), the X-ray beam is collimated and monochromated, as demonstrated in the DuMond diagram shown in Fig. 7 ▶. The resolution element is given by the parallelogram 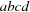. In this case the beam divergence equals the Darwin width 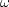. Rocking the crystal 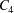, which is in a non-dispersive arrangement with the crystal 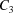, should provide quantitative information about the perfection of the crystals 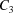 and 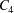.

The performance of the Si(511) delay line optics was tested with an undulator source of vertical divergence 17 µrad. The incident beam was monochromated by Si(111) and Si(333) channel-cut monochromators. The delay line was operated in a configuration with four Bragg crystals, in which the beam splitter 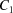 was replaced by the Bragg crystal 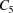. Measured data were normalized to the monitor count rate. Since the experiment was performed under ambient conditions, the measured rocking curves were corrected for absorption losses.

**Figure 8:**
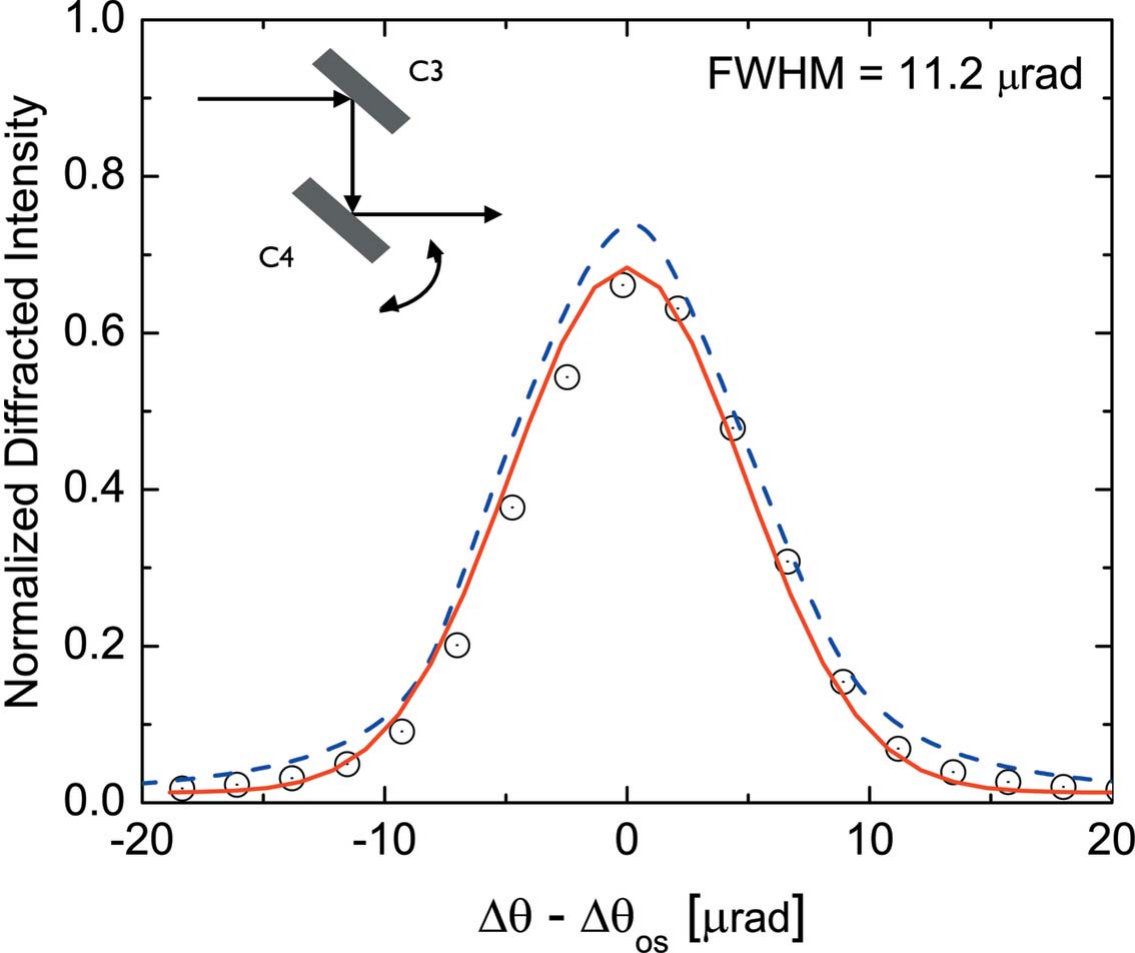
Measured reflectivity of Si(511) in symmetric Bragg geometry at *E* = 8.39 keV. 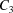 and 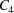 denote the positions of the Bragg crystals depicted in Fig. 1 ▶. The result of a Gaussian fit to the data and the calculated rocking curve is shown by the solid red and dashed blue lines, respectively.

Fig. 8 ▶ shows the rocking curve of the Bragg crystal 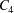. The inset shows the schematic set-up. The resulting curve is a convolution of two Darwin curves of the crystals 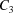 and 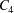. Therefore, it contains information about the quality of both crystals. The red solid line in Fig. 8 ▶ is the result of a Gaussian fit to the experimental data. The expected rocking curve is depicted by the blue dashed line yielding a peak reflectivity *R* = 0.73 and a width of 12.2 µrad. The observed FWHM = 11.2 µrad and *R* = 0.65 are in a good agreement with the expected values, indicating very good quality of the two Bragg crystals. All delay line crystals were cut from the same silicon ingot and all measured crystals are of similar quality.

### 4.2. Delay line beam splitter

**Figure 9:**
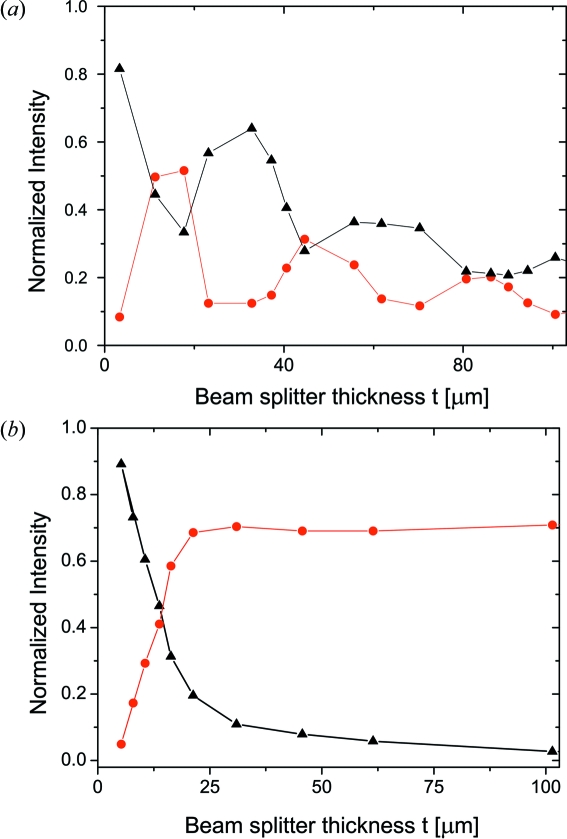
Variations of the diffracted intensities in the case of a Laue (*a*) and Bragg (*b*) beam-splitter crystal. Circles and triangles denote the intensity of the Bragg-reflected and forward-diffracted beams, respectively.

The performance of Si(511) Laue and Bragg beam splitters has been investigated. The incident beam divergence was reduced by primary and exit slits set to 100 × 100 µm, located 19.3 m and 21.65 m from the source, respectively. Furthermore, the beam was monochromated using Ge(111) and Si(333) perfect crystals. The intensities of the diffracted beams were collected by a detector placed above and downstream of the splitter on the direct beam path and normalized to the incident number of photons. The wedge-shaped crystal was probed at various positions. Fig. 9(*a*) ▶ shows a plot of the intensities diffracted by the Laue beam splitter. The data were measured at the angular position where the Bragg angle is exactly satisfied, *i.e.* 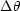 = 0, as a function of crystal thickness 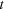. A splitting ratio close to 1:1 was achieved by using the crystal at a thickness of 11.2 µm. At this position 50% of the beam is reflected and 45% is diffracted in the forward direction. About 5% of the intensity is lost due to absorption. One can note that a splitting ratio of 1:1 can also be obtained at a thicker part of the crystal, *i.e.* 28 µm, 86 µm or 116 µm. At these crystal positions the Pendellösung oscillations are blurred owing to the unequal absorption of the wavefields in the crystal (Ishikawa, 1988 ▶). This effect reduces the stability demands on the incident beam at the expense of increased absorption by more than 40%. Since the delay line employs two beam splitters the absorption losses need to be minimized by working at the thinner (below 20 µm) part of the crystal. The largest splitting ratio (*i.e.* close to 1:5) is observed by probing the crystal at the thickness 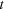 = 32.8 µm. At this crystal position only 12% of the beam was reflected and 64% was diffracted with the momentum parallel to the incoming beam.

Fig. 9(*b*) ▶ shows the variation of the intensities diffracted by the Bragg beam splitter. One can clearly notice the absence of Pendellösung oscillations. The intensity of the reflected beam gradually increases as the crystal is probed at the thickness ranges from 4 up to 20 µm. The ratio of 1:1 splitting was observed at a thickness of 14 µm instead of the theoretically expected 6 µm. For *t* > 20 µm the intensity is reflected with an efficiency of 76%. In this case the crystal acts as a Bragg mirror for 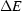 = 77 meV centered at *E* = 8.39 keV. The radiation outside the Bragg bandwidth can still be transmitted through the crystal with 77% efficiency.

### 4.3. Throughput of the delay line

The performance of the Si(511) delay unit optics with the Laue beam splitter and mixer crystals has been tested in the two-branch configuration. To optimize the throughput, all crystals were aligned to provide maximum reflectivity. The throughput 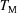 was obtained from the ratio of the X-ray intensity measured by the APD detector located upstream and downstream of the delay line. The results of the throughput measurements are summarized in Table 2 ▶.

The throughput obtained using a Si(333) monochromator located upstream of the delay line is 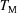 = 6 × 10^−3^. The lattice spacing of the Si(333) and Si(511) reflections are the same and thus the Si(333) monochromator and Si(511) beam splitter constitute the non-dispersive geometry. In addition, the incident radiation was well collimated by slits located upstream of the delay line. Therefore, the transmitted flux measured in this configuration can be mainly attributed to the performance of the delay line optics. The calculated values of the throughput [based on ray tracing (Seeck, 2006 ▶) and including absorption] are in agreement with the measured values. The transmission value of the delay line optics measured with a Si(111) pre-monochromator is 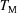 = 2.7 × 10^−4^. Since the wavelength bandpass of a Si(111) monochromator is 16 times larger than that for Si(333), one expects in this case a smaller value for the delay line throughput.

A higher throughput is expected for Bragg beam splitters. The X-ray beam splitter in Laue geometry disperses wavelengths into angles owing to inherent chromatic aberration (Brauer *et al.*, 1995 ▶). Any increase of the beam divergence, introduced by the first beam splitter 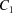, is filtered by the (+*n*, +*n*) configuration of the crystals 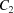 and 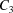, leading to a decrease of the overall throughput.

### 4.4. Stability of the delay line

**Figure 10:**
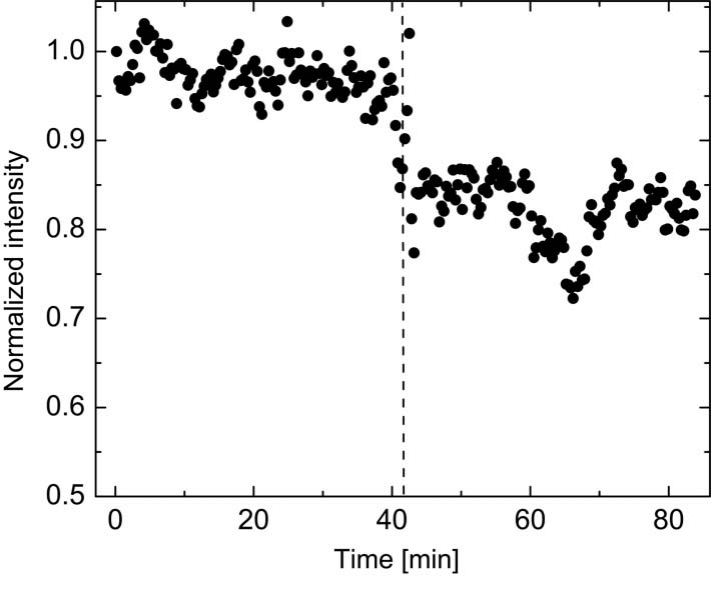
Stability of the Si(511) delay line optics at *E* = 8.39 keV. The vertical dashed line indicates a refill in the ESRF storage ring.

The stability of the delay line components is essential for conducting pump–probe and correlation spectroscopy experiments. Both aforementioned techniques will be performed in a stroboscopic manner. Therefore, the device should provide a stability such that no crystal realignment is necessary during the acquisition time for a given delay. Fig. 10 ▶ shows the intensity measured by the APD detector, located downstream of the delay line. The data were corrected for the variations of the storage ring current and normalized to the value of the intensity recorded at time 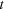 = 0. The device can be operated without realignment for typically 30 min. An abrupt jump of the intensity, recorded after 40 min, was observed and is related to a refill of the ESRF storage ring. After the refill, 83% of the intensity is recovered without realignment. Neither temperature stabilization nor active feedback was used in the set-up.

## 5. Delay time measurements

**Figure 11:**
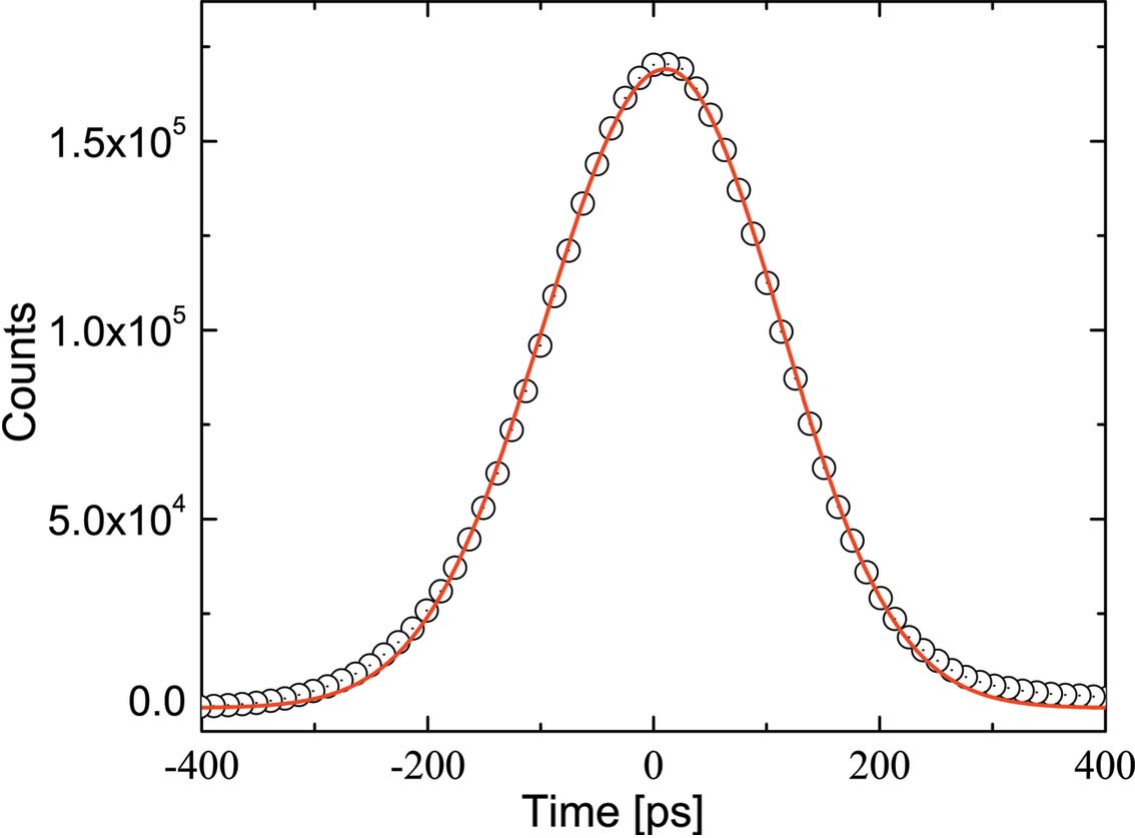
Temporal response of the ultra-fast APD detector to 8.39 keV radiation measured at ID10C. The solid red line is a fit of equation (5) to the data and yields 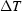 = 235 ps.

In order to evaluate the time resolution of the delay line the timing performance of the APD detector has been tested prior to the experiments. Fig. 11 ▶ shows the resulting time response pattern of the detector during four-bunch operation of ESRF. Since the measured pattern shows a slight asymmetry, the peak position 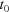 and its maximum intensity 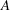 is extracted by using an asymmetric Gaussian function (Kato *et al.*, 2002 ▶) defined by
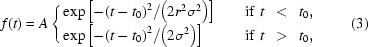
where 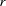 is the asymmetry parameter. The standard deviation 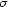 in (3) is related to time resolution 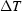 (FWHM) according to
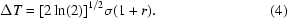
For an asymmetry parameter 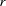 = 1, equation (4) simplifies to a symmetric Gaussian function and 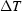 = 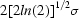.

The solid line in Fig. 11 ▶ is the result of the fit of (3) to the measured time pattern which yields *r* = 0.95 and 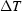 = 235 ps. The latter value contains contributions from the length of the electron bunch 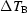 and the detection system (*i.e.* APD detector, bunch clock signal and CFD/TAC/ADC electronics shown in Fig. 6 ▶), *i.e.* 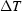 = 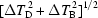. *Via* deconvolution the resolution of a detection system is 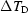 = 189 ps with 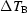 = 140 ps.

**Figure 12:**
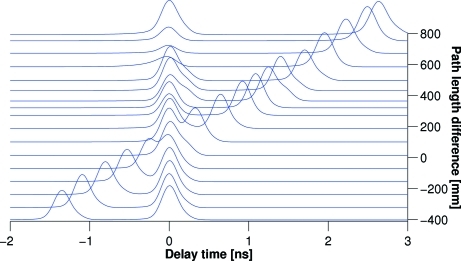
Time patterns corresponding to delayed X-ray pulses measured as a function of 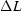. The maximum measured 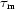 is 2.63 ns.

Fig. 12 ▶ shows a plot of the time patterns recorded at various settings of the delay line. Since photons are delayed by the upper and the lower branch, the measured time pattern reveals two peaks. In order to obtain statistically significant data, each time pattern was measured over approximately 30 min. For the sake of clarity the position of the X-ray pulse delayed by the lower branch of the delay line was offset to *t* = 0. Each time spectrum was normalized to the value of the maximum peak value. The shape and the peak intensity varies for the first four top time delay patterns (corresponding to the longest delay times). The peak shape can be influenced by the detector electronics. The peak intensity variation is due to a non-ideal alignment procedure of the delay line. The delay time between the peaks is obtained by a fit of two asymmetric Gaussian functions to the data [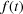 = 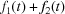, where 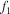 and 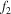 are given by (3)]. The measured delay time 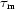 is then simply the difference between the positions of the two measured peaks. Since both pulses are delayed with respect to the direct beam the measured 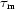 gives a relative delay time. Obtaining the absolute value of the delay time, which is of importance for laser pump–X-ray probe type of experiments, requires a measurement of the direct beam.

**Figure 13:**
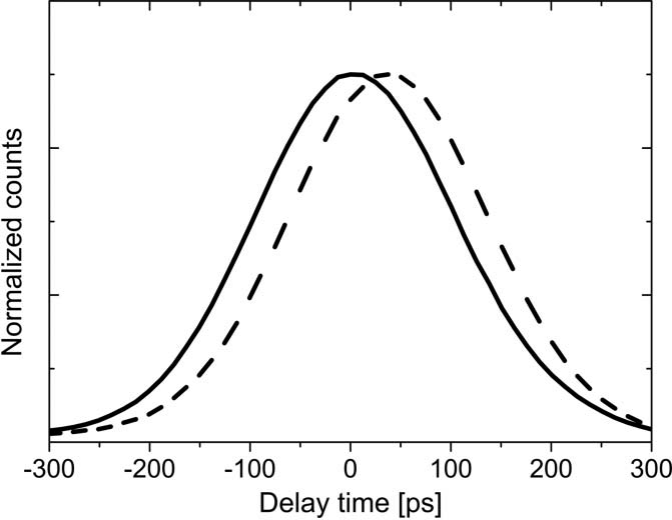
Two time patterns recorded when either the upper (solid curve) or lower (dashed curve) branch of the delay line was blocked. The delay time 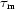 is 36 ps.

At a path length difference 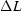 of 792.75 mm the time pattern reveals two equally intense X-ray pulses separated by 2.626 ± 0.003 ns, which corresponds to the maximum delay time 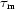 achieved in the two-branch configuration. Delay times up to 2.95 ns have been measured with the single branch. Delay times smaller than the duration of the electron bunch can be measured by the set-up as well. However, in this case it is required to record time patterns separately for each delay line branch, as shown in Fig. 13 ▶.

**Figure 14:**
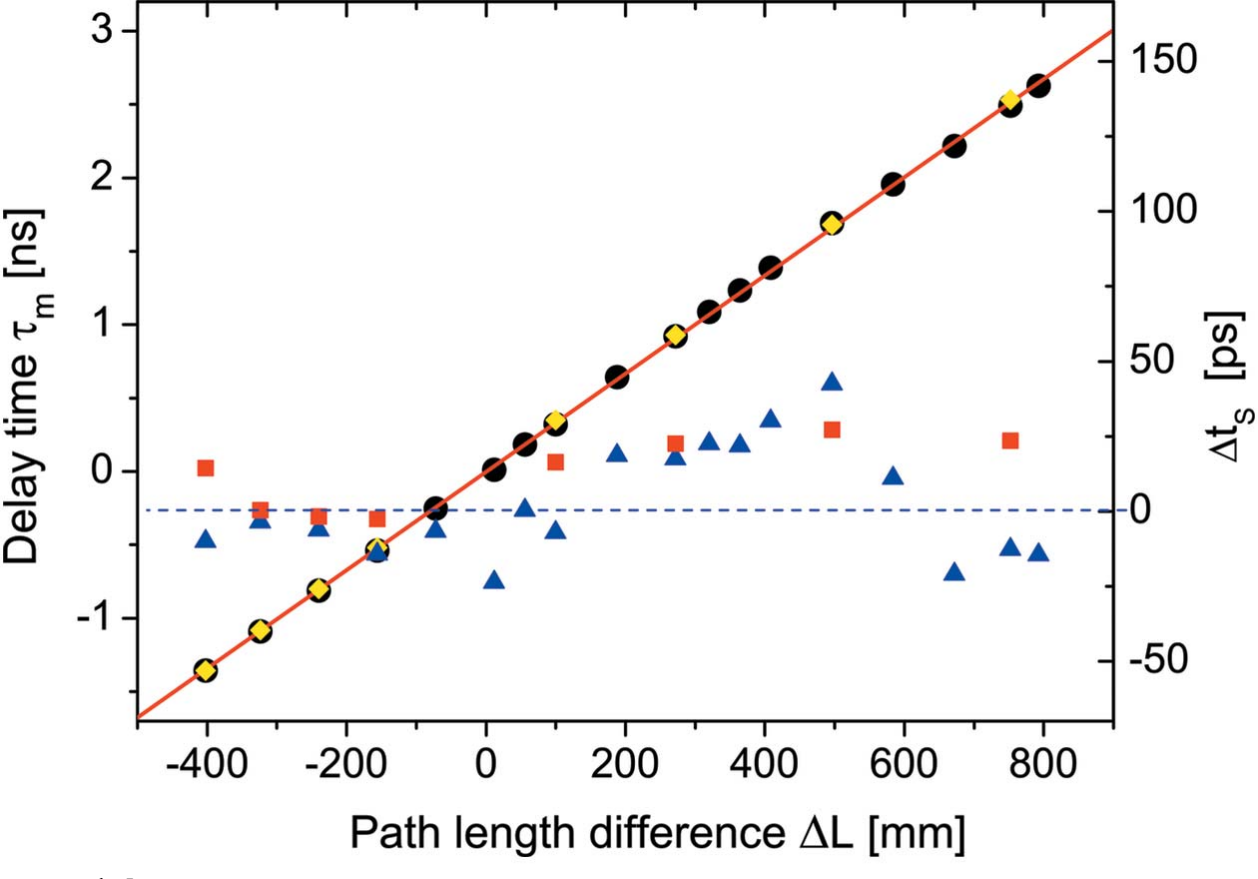
Measured delay time 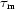 *versus* applied path difference 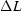 (left axis). Black and yellow circles correspond to two sets of delay time measurements. The solid red line is a linear fit to the data (black circles). The difference between the measured and calculated delay time is denoted by the blue triangles and red squares (right axis).

Fig. 14 ▶ shows a plot of the measured delay time 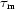 as a function of the path length difference 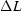. The (solid) red line is a least-squares fit to the data. It yields a slope of 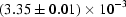
 ns mm^−1^ and an offset of −4.3 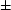 3.2 ps. The delay time increases linearly with the path length 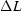 indicating a very high precision of the mechanics and promises an operation without the need for higher-order correction terms when setting the path length difference. The delay time error 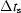 = 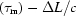 was extracted for every data point. The results (corrected for the offset) are also shown (blue triangles) in Fig. 14 ▶. The mean delay time error is 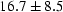
 ps. The maximum deviation was found to be less than 43 ps. The results of the second set of delay time measurements are denoted by yellow diamond symbols in Fig. 14 ▶. The averaged mean error of the two measurements is 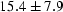
 ps.

The time resolution achieved in the measurements does not represent the intrinsic limit of the delay line. It is mostly given by the detection system of the delay line, shown in Fig. 6 ▶. The limits for the intrinsic time resolution of the current set-up are given by the mechanical components and the temperature stability of the delay line. The positional accuracy of the central translation unit is 0.5 µm, which allows the delay time to be changed with a precision of not better than 7 fs. Since the delay line operates under ambient conditions, a change in temperature affects the position of the delay line crystals owing to the thermal expansion of the main aluminium plate. The expected effective change in the measured delay time 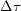 is 100 fs for a temperature fluctuation of 1 K.

## 6. Experiments with coherent X-ray beam

**Figure 15:**
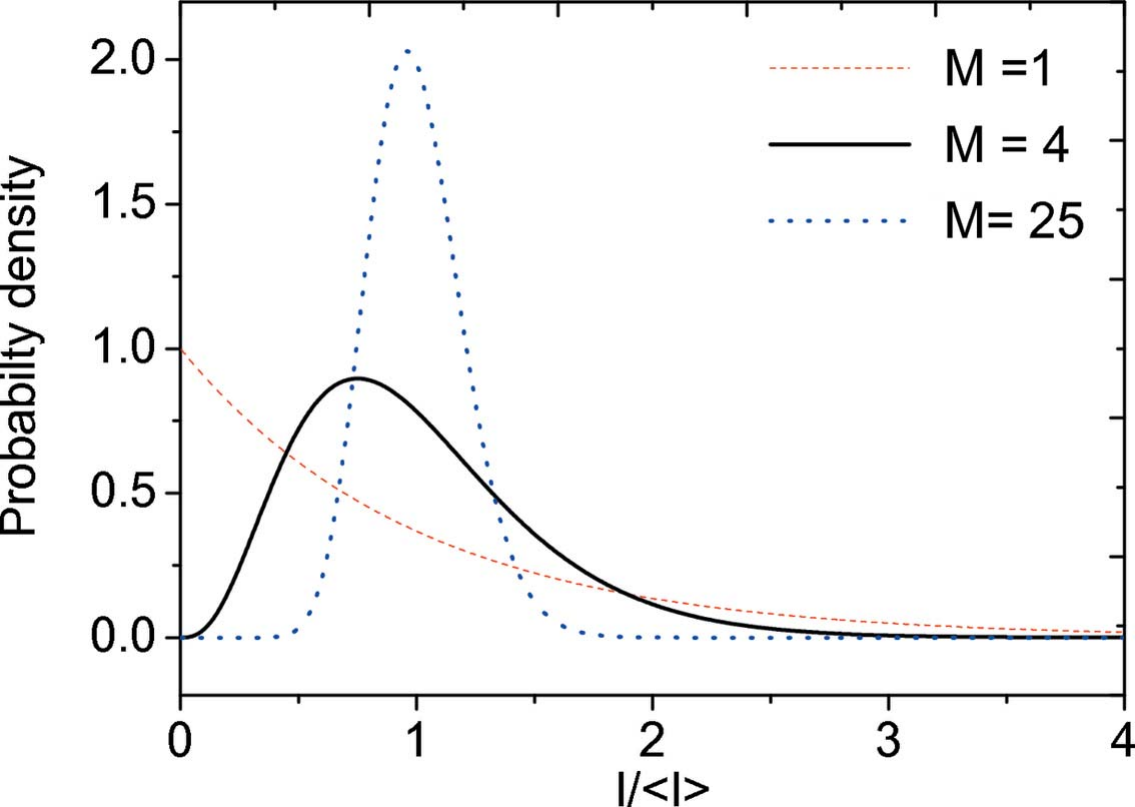
The probability density 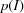 of photons of a fully coherent (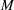 = 1) beam. The effect of partially coherent illumination is demonstrated by plots with 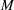 = 4 and 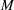 = 25.

A necessary condition for performing XPCS experiments is coherent illumination of the investigated sample. More specifically, the illuminated area of the sample has to be smaller than the coherence area, which is determined by the product of horizontal and vertical transverse coherence lengths. In addition, the path length difference (PLD) of X-rays in the sample has to be smaller or comparable with the longitudinal coherence length. Coherent X-rays that are scattered from the sample give rise to speckles in the far field detection plane. The resulting pattern reflects the exact spatial arrangement of the scatterers in the sample. The intensity probability distribution 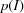 of a speckle pattern is a measure of the coherence properties of the beam and can be used to monitor any degradation of the beam properties caused by imperfect optics. For a fully coherent beam,
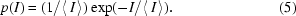
For a partially coherent beam the observed speckle pattern can be described as a sum of *M* coherent speckle patterns, with contrast *C* = 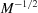 (Goodman, 2006 ▶). In this case the probability distribution 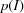 is given by
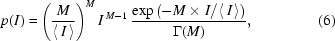
where 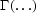 is the gamma function. Fig. 15 ▶ shows the probability distribution function plotted for different values of 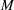. For *M* = 1, equation (6) simplifies to (5), which reflects the intensity distribution of the fully developed speckle pattern and indicates the conditions of fully coherent illumination. In the case of incoherent illumination, 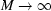 and the probability distribution approaches a delta function centered at the mean value of the intensity 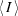.

The presence of optical components such as lenses, windows or monochromators in the beam path can degrade the degree of coherence of the beam causing a reduction of the contrast of the speckle pattern in the XPCS measurement. Therefore the knowledge of the influence of the delay line on the coherence properties of undulator radiation was investigated prior to performing XPCS experiments at an XFEL source.

The static speckle pattern experiments were carried out at the ID10C undulator source. The two-branch configuration of the delay line with the Laue beam splitters was used. The final size of the X-ray beam was defined by a pair of roller blade slits (Le Bolloc’h *et al.*, 2002 ▶) located downstream of the delay line at 63 m from the source. At this position the expected horizontal and vertical transverse coherence lengths are 16 µm and 202 µm, respectively. The delay time was set to 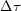 = 273 ps, which is much longer than the longitudinal coherence time (*i.e.* 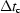 = 1.27 ps). Therefore, the resulting speckle pattern is not affected by interference of the two beams passing through the delay line. A static sample, SiO_2_ powder, was placed in a 0.5 mm-thick capillary in a custom-designed small-angle X-ray scattering (SAXS) chamber (Robert, 2001 ▶), which was located 155 mm downstream of the roller blade slits. The static speckle patterns were recorded by a direct-illumination CCD (Princeton Instrument, LCX) camera which comprised 1340 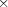 1300 pixels, each of dimension 20 µm 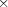 20 µm.

**Figure 16:**
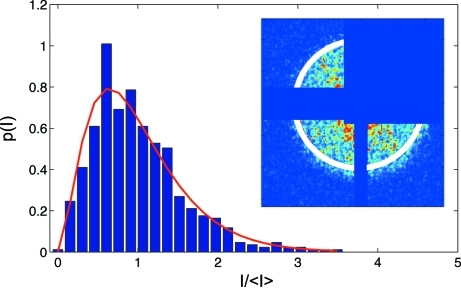
Probability density of the speckle pattern recorded with the delayed beam. Inset: corresponding speckle pattern. The area denoted by a white circle corresponds to the analyzed scattering vector of 2 × 10^−3^ Å^−1^.

Fig. 16 ▶ shows the speckle pattern recorded downstream of the delay line. The sample was illuminated with a beam size of 10 µm 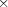 10 µm. Since the nominal vertical and horizontal transverse coherence lengths are larger at this position, the aperture should select a coherent portion of the incident radiation. In this configuration the expected coherent flux at the sample position is 2.2 × 10^3^ photons s^−1^ at 40 mA ring current. This value is more than five orders of magnitude lower than typically reported for experiments dedicated to statistical speckle analysis (Abernathy *et al.*, 1998 ▶). The image shown in Fig. 16 (inset) ▶ was obtained by summing 100 × 60 s single exposures. The electronic noise of the CCD was accounted for in the analysis by measuring a series of 60 s dark images (*i.e.* without X-ray beam) and its average was subtracted from each frame before summation. Recorded intensities [in analog-to-digital units (ADU)] were converted into photon numbers. The number of 750 ADU per detected photon was determined using the droplet algorithm (Livet *et al.*, 2000 ▶). The speckle size was matched to the pixel size by placing the detector 2.45 m downstream of the sample. The inset of Fig. 16 ▶ shows the speckle pattern. The expected speckle size in this configuration is 35 µm. Parasitic slit scattering was present at the detection plane, causing a distortion of the speckle pattern. The area corresponding to the beam stop and the slit scattering was masked, as illustrated in Fig. 16 ▶, and not taken into account during the data analysis.

The small-angle scattering signal recorded from the sample was analyzed. The data were averaged over annuli of constant scattering wavevector *q* = 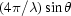, where 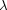 and 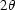 are the wavelength and the scattering angle, respectively. The intensity of the averaged signal falls off rapidly with increasing 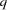 and shows modulations as expected for a sample consisting of monodisperse spherical particles. The small-angle scattering profile was fit with a model utilizing the form factor of spherical particles convoluted with a Schulz size distribution function. The fitting procedure yields an average radius *R* of 1699 Å.

Fig. 16 ▶ shows the probability density function of the speckle pattern displayed in the inset of Fig. 16 ▶. The intensities in the 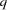 ring from 0.0019 Å^−1^ to 0.0021 Å^−1^, denoted by the white circle, were selected and normalized to the mean intensity 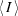. The probability density function, 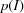, was obtained by histogramming the selected intensities. The obtained histogram shows a wide distribution of intensities, which is a characteristic feature of a speckle pattern.

The solid line of Fig. 16 ▶ shows a fit of equation (6) to the experimental data. The fit procedure yields *M* = 3.2 ± 0.4, which corresponds to a contrast *C* = 0.56 ± 0.04.

**Figure 17:**
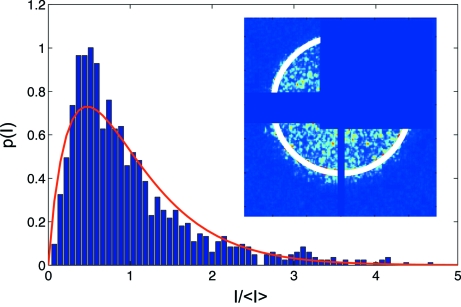
Probability density of the speckle pattern recorded with the direct beam. Inset: corresponding speckle pattern. Photons recorded at scattering vector 2 × 10^−3^ Å^−1^ were selected in the analysis and are denoted by a white circle.

In order to evaluate the influence of upstream beamline components on the speckle pattern contrast, the same analysis at the same 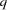 value was applied to the speckle pattern obtained with the direct beam, *i.e.* when the delay line was removed from the beam path. Fig. 17 ▶ shows the resulting speckle pattern and the corresponding probability density function. The fit procedure yields *M* = 2.1 ± 0.2 and a contrast of *C* = 0.69 ± 1 0.04, a value 13% higher than with the delay line, which is interpreted as an effect introduced by the optical components inserted into the beam path. Such a moderate effect of coherence degradation indicates the feasibility of performing coherent scattering experiments with the delay line.

## 7. Summary and conclusions

We have demonstrated the successful implementation of the 90° scattering geometry for the delay line concept at 8.39 keV. The device employs up to eight perfect silicon crystals that split a single X-ray pulse into two sub-pulses. The two pulses propagate through two branches of the delay line before they are recombined by the beam mixer. The beam splitting and mixing is accomplished with Laue crystals and the X-ray path inside the delay line is defined by Bragg crystals. The quality of the Si(511) crystals was verified by rocking-curve measurements. The results yield rocking-curve widths in agreement with the theoretical values, showing the high quality of the silicon crystals. The performance of the X-ray beam splitters was also tested. Splitting ratios from 1:1 to 1:5 were achieved. A throughput of 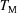 = 0.6%, which is very close to the expected value, is achievable under ambient conditions. Delay times up to 2.63 ns and 2.95 ns have been achieved with the two-branch and single-branch configuration, respectively. A time resolution of 15.4 ps was achieved in the measurements. This value is mainly determined by the time resolution of the detection system. Higher time resolution should be achievable by using, for example, a streak camera or laser interferometry. The influence of the delay line on the coherence properties of the undulator source was investigated by analyzing spatial intensity fluctuations of static speckle patterns. A reduction of the contrast by 13% is observed. The device is being implemented at the LCLS/XPP at SLAC. First experiments will be carried out in January 2011.                

## Figures and Tables

**Table 1:** Energy 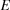, Darwin width 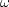 and reflectivity 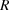 of various silicon reflections at the dynamical Bragg angle of 45° in symmetric scattering geometry

*E* (keV)	*hkl*	ω (µrad)	*R*
8.39	333	8.6	0.89
	511	8.6	0.89
9.13	440	9.3	0.96
9.55	531	5.7	0.91
10.59	533	4.1	0.91
11.18	444	4.8	0.96
11.53	551	3.1	0.91
	711	3.1	0.91
12.40	553	2.3	0.92
	731	2.3	0.91
12.91	800	2.8	0.95

**Table 2:** Measured 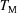 and calculated 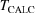 throughtput of the delay line obtained with Si(111) and Si(333) pre-monochromators at *E* = 8.39 keV. 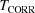 denotes the measured throughput values corrected for the X-ray absorption in air

	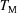	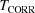	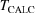
Si(333)	6.0 × 10^−3^	6.6 × 10^−2^	6.8 × 10^−2^
Si(111)	2.7 × 10^−4^	3 × 10^−3^	2.9 × 10^−3^

## Footnotes

1
                is not limited by the minimum separation between optical components, *e.g.* the crystals  and .

2 Calculated using *XOP* software (Dejus & Sanchez del Rio, 1996 ▶).

3 The value achieved using the micro-stepping and the Renishaw RGH24 encoder system.
